# War and Insanity

**Published:** 1914-08-08

**Authors:** 


### The Hospital

The Workers' Newspaper of Administrative Medicine and Institutional Life, Administration, National Insurance and Health.

No. 1468. Vol. LVI. SATURDAY, AUGUST 8, 1914. PRICE ONE PENNY.

### WAR AND INSANITY.

The history of the world proves that war has often had its origin in insanity. Last week we wrote on "The Decay of Moral Force" and its effects upon the character of the British race. Since that article appeared, Sir Edward Grey has made history by his masterly statement in the House of Commons on the 3rd instant. This utterance was probably the most forcible which has been made in the House of Commons for decades of years. Its force is due to demonstrable facts in sequence which have shattered the masks and falsehoods put forward by enemies with the object of imposing upon the world. Any further attempts to deceive mankind, after Sir Edward Grey's statement, must fail.

The decay of moral force has been a marked feature of recent times in many nations. The evidence laid before the House of Commons by the Government on the 3rd and 4th instant demonstrates once more that madness may precede destruction when the gods take action. Is it conceivable otherwise that any man of intelligence and authority could persuade himself that he had behind him sufficient money, weapons of destruction, and fists, to compel the whole world, and the people it contains, to fall down and worship him at his will and pleasure? Yet this belief seems to dominate some of the contestants at any rate, or they could never have brought themselves publicly to shed all the high standards of conduct, religious sentiment, and principles which they have heretofore publicly claimed to possess.

What is the evidence the war presents to the scientific world whose medical members are responsible for certifying the sanity or madness of individuals? In the present case what the Tsar of Russia properly defines as "a mean war," was started in a hurry by a Great Power with a small kinglet. When the sober minds governing the world attempted to limit the area of the conflict and to exclude from it most of the Great Powers of Europe, the patient grew first restless and then crafty. Restless because of the fear of being thwarted in his purpose, and crafty in claiming to work for restricting the conflict to a narrow area, whilst he was hastening his fevered preparations for war. So vigorous and sincere were the efforts for restriction and peace that they would have been entirely successful if allowed to continue for a few days longer. Seeing this the patient, in his haste to capture the world, declared war upon his chief neighbour directly he consented to negotiate for peace. This done, every possible device and insult was resorted to to bring first one Great Power and then another into the strife. When these attempts failed he declared war against them all. But our patient could not be content! He had got a plan of what he wished to do and how it could be made successful. He accordingly broke the honourable engagements into which he had entered, tore up the documents, and proceeded to bring himself into a state of war with small and great. Indeed, it seems, as we write, that our patient has succeeded in creating Armageddon. The civilized world, first shocked and horrified, is now uniting, and has placed the patient under strict observation. There can be no doubt that any war thus brought about is not only a mean war, but a war originating in madness. The world must realise at the outset, by the facts now known to all the peoples, that the real authors of the present European war, their objects, actions, and ambitions, afford evidence to justify a properly constituted Board of Lunacy in placing them in strict confinement for their own safety and that of other people.

The present war in its origin rests upon moral degeneracy and an absence of sound judgment. Its authors have persuaded themselves that the money they have accumulated, the armaments and munitions of war which they have created, and the control of the enormous armies they have drilled to the use of these implements of destruction, will prove irresistible. A lunatic always believes that he is irresistible and not infrequently claims to be the Deity himself. It will be plain to all thoughtful minds that the nations of the earth should combine and quickly stamp out this war by placing the authors of it in safe confinement, out of mischief. They can never be permitted to control, to rule and to enslave the world.

Three Great Powers, and several small ones, have had war declared against them by the madmen who are responsible for the present threatened Armageddon. One Great European Power, to its infinite credit, still stands neutral, and there are also the United States of America, who have no direct interest in the causes which underlie the present troubles. Put very shortly, a relatively few madmen, fired by inordinate greed and ambitions, have, for their own purposes, converted Europe into a huge battlefield. When the civilized nations of the earth realise this to be so, it seems a certainty that every country possessed of a sane Government will spontaneously come forward and join hands with other resisting peoples with the view of stopping the demented and guilty beings responsible. Civilization demands the unity of civilized nations to guarantee the sober government of the world and the defeat of insanity.

